# Neurological Assessment Using a Quantitative Sensory Test in Patients with Chronic Unilateral Orofacial Pain

**DOI:** 10.2174/1874210601812010053

**Published:** 2018-01-31

**Authors:** Talal H Salame, Antony Blinkhorn, Zahra Karami

**Affiliations:** 1 Department of Prosthodontics, Faculty of Dentistry, The Lebanese University, Rafic Harriri Campus, Hadath, Lebanon; 2 Department of Population Oral Health, Faculty of Dentistry, The University of Sydney, New South Wales, Australia; 3 Department of Oral Rehabilitation, Faculty of Dentistry, The University of Sydney, New South Wales, Australia

**Keywords:** QST (Quantitative sensory test), Neurological assessment, Orofacial pain, Chronic pain diagnosis, Warm sensation, Cold sensation

## Abstract

**Background:**

Quantitative Sensory Testing (QST) has been used in clinical and experimental settings to establish sensory assessment for different types of pains, and may be a useful tool for the assessment of orofacial pain, but this premise needs to be tested.

**Objective:**

The aim of the study was to evaluate responses to thermal stimuli between painful and non-painful facial sites in subjects with orofacial pain using QST.

**Methods:**

A total of 60 participants (5o females: 28-83 years; 10 males: 44-81 years) with unilateral orofacial pain were recruited from the Orofacial Pain Clinic at the Pain Management and Research Centre, Royal North Shore Hospital, Sydney, Australia. The study followed the methods of limits of the German Research Network testing four modalities of thermal thresholds, the Warm Sensation, the Cold Sensation, the Heat Pain and the Cold Pain using a TSA-II Neurosensory Analyser. The results were compared to the results from the unaffected side of the same patient on the same area and a single t test statistical analysis was performed, where a p value of less than 0.05 was considered significant.

**Results:**

The Mean Difference for Cold Sensation between the pain side and the non-pain side was 0.48 °C ± 1.5 (t= 2.466, p=0.017), 0.68 °C ± 2.04 for Warm Sensation (t= -2.573, p= 0.013), 2.56 °C ± 2.74 for Cold Pain (t= 7.238, p<0.001) and -1.21 °C ± 2.59 for Hot Pain (t= -3.639, p=0.001).

**Conclusion:**

The study showed that QST methods using thermal stimuli could be used to evaluate sensory dysfunction in orofacial pain patients using the specific parameters of cool and warm sensation, and cold and hot pain.

## 1 INTRODUCTION

Pain assessment in the context of a reaction to an impulse of temperature using a computerised thermoset, such as Quantitative Sensory Testing (QST) has been used effectively over the past two decades as a reliable method for detecting and quantifying positive and negative sensory phenomena in different types of neuropathies and chronic pain conditions. It is used to measure reactions to typical thermal or mechanical stimuli in patients with neuropathic pain, to identify pain thresholds subsequent to induction of rising strength [1, 2], and to identify the pathophysiological mechanisms present in certain types of neuropathic pains. QST has also been used to examine deep pain and cutaneous sensitivity to generate sensory sign profiles for thermal and mechanical stimuli [3-7].

The German Research Network on neuropathic Pain (DFNS) has developed a standardized QST protocol in which cold detection threshold (CDT), warm detection threshold (WDT), cold pain threshold (CPT) and heat pain threshold (HPT) can be measured following the method of limits [5]. A thermal sensory limen procedure (TSL) was performed in between to determine the paradoxical heat sensations (PHS) for alternating cold and warm stimuli. It should be emphasized that alterations in QST were also identified in non-neuropathic pain conditions, such as rheumatoid arthritis, inflammatory arthromyalgias and fibromyalgia [8-10].

QST has not been widely used in patients with orofacial pain conditions and it may be of value to test this tool for the assessment of orofacial pain. Therefore, the aim of the study was to evaluate responses to thermal stimuli in subjects with orofacial pain, specifically the response between painful and non-painful facial sites, according to the German Research Network on neuropathic pain.

## 2 MATERIALS AND METHODS

**Fig. (1):**
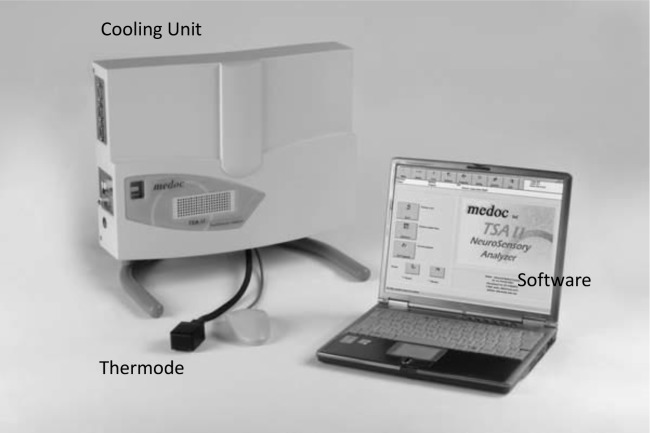
TSA II NeuroSensory Analyser - Software, Cooling Unit and Thermode. (*Photo is taken from TSA-II USER and SERVICE GUIDE 2010*).

A total of 60 participants suffering from unilateral orofacial pain were recruited from patients who attended a weekly Orofacial Pain Clinic at the Pain Management and Research Centre, Royal North Shore Hospital, Sydney, New South Wales, Australia, by the supervising consultant. Pain is supposed to be chronic (*i.e.* occurring or recurring for the last 6 months or more), not contributed to a known systemic disease (*e.g*. osteoarthritis, cancer pain), not resultant from a trauma, and not diagnosed as psychosomatic. Of these 60 subjects fifty were female (mean age 55.3 years, SD= 12.14, range 28-83 years) and ten male (mean age 64.4 years, SD= 13.52, range 44-81 years). Subjects were excluded if they had a history of psychiatric or another illness in which medications (*e.g.* anticonvulsants, antidepressants, and analgesics) were taken that could affect the pain response. The project was approved by the Human Research Ethics Committee (HREC) of Northern Sydney Central Coast Area Health (NSCCH) and the Human Research Ethics Committee of The University of Sydney. The instrument used to collect the research data was a TSA-II Neurosensory Analyser (Fig. **1**). This device is controlled by designated software that can produce repeatable thermal stimuli [11, 12].

The study followed the protocol of the German Research Network (DFNS) [5], using the methods of limits [12] and testing four modalities of thermal thresholds:

- Warm sensation – (1-2°C above 32°C) to assess C-fibre mediated response
- Cold sensation – (1-2°C below 32°C) to assess A-delta fibre mediated response
- Heat induced pain – (around 45°C) to assess predominantly C-fibre mediated (with some A-delta fibre) response
- Cold induced pain – to assess a combination of both C and A-delta fibre mediated response.

In the method of limits, the intensity of stimulus changes until it is halted by the patient when the required sensation is felt. The thermode temperature then returns to the adaptation temperature for the next stimulus.

Each participant was given a brief demonstration and instructed to remain still and hold the Thermode (1.6 x 1.6 cm) over the centre of the painful site (*i.e*. where pain usually begins if the area is touched) or on the area where pain commences. The test starts when the temperature control unit achieves the requested temperature (baseline temperature initially determined by the investigator). The temperature baseline varies between 30°C and 32°C and it is determined when the subject feels neither warmth nor cold after a few seconds of skin contact with the Thermode.

The Cold sensation (CS) and warm sensation (WS) rate of temperature change was set at 1°C/second. The cold-induced pain (CP) and heat-induced pain (HP) rate of temperature change was set at 1.5°C/second. The interval between stimuli (from the end of one stimulus to the onset of the next stimulus) was set at 4-6 sec. in CS and WS and 10 sec. in HP and CP.

Participants were instructed to click the mouse of the computer at exactly the moment they first felt the temperature change (in the case of thermal sensation) or as soon as they felt their pain threshold had been reached (in the case of cold pain/hot pain) and they were informed that a prompt reaction to the temperature change was very important. As soon as they pressed the mouse, the Thermode temperature immediately returned to the baseline temperature for the next stimulus. Four different clusters of stimuli were given, with five stimuli in each cluster (a maximum of 20 stimuli in a test). The whole or part of the test was repeated if the participant inadvertently pressed the key too early or too late.

The 60 participants were diagnosed with different types of pain conditions and they were all grouped together as unilateral orofacial pain patients. A total of 18 trials were recorded on each side of the face. The mean value of the set of experiments for each modality (CS, WS, CP, and HP) was then calculated and a single t test statistical analysis was performed, where a *p* value of less than 0.05 was considered to be significant (*SPSS* 20 statistical package).

## 3 RESULTS

The test results were compared to the results collected from the unaffected side of the same patient on the same area on the other (control) side of the face. Differences in the test results between one side of the face and the other may indicate peripheral nerve disease or injury.

As shown in Table **1**, the Mean Difference for Cold Sensation between the pain side and the non-pain side was 0.48 °C ± 1.5, 0.68°C ± 2.04 for Warm Sensation, 2.56°C ± 2.74 for Cold Pain and -1.21°C ± 2.59 for Hot Pain. Fig. (**2**) shows that there was a statistically significant difference between the pain side and the non-pain side for Cold Sensation (t= 2.466, *p*=0.017), for Warm Sensation (t= -2.573, *p*= 0.013), for Cold Pain (t= 7.238, *p*<0.001) and for Hot Pain (t= -3.639, *p*=0.001).

## 4 DISCUSSION

This study has shown that participants with unilateral orofacial pain exhibited dysfunction of thermal processing on the pain side compared to the non-pain side. These findings are similar to other studies that have used QST and showed disruption of thermal processing in patients with different types of chronic pain conditions [13-18]. For instance, Launtenbachter and colleagues [14] tested thermal perception in 26 female patients with fibromyalgia. They compared their results within patients, between pain sites and non-pain sites, and with controls. Similar to our study, they found significant differences in pain patients between the pain site and the non-pain site. Also, there were significant differences in thermal perception between both sites and mirror sides in non-pain volunteers. Kosek and Orderberg [17] performed a QST study on patients with osteoarthritis affecting their hips and they also found significant alteration of thermal sensitivity in their results. Ochoa and coworkers [18] found that thermal sensations were abnormal in all their neuropathic pain patients. They also used QST methods and as in this study all 4 modalities (WS, CS, HPT and CPT) were tested. Similarly, in their two studies using same QST protocol in patients with fibromyalgia, Kosek and coworkers [15, 16] found alteration of thermal perception in these patients. These findings support our results and suggest that patients with varying conditions of chronic pain exhibited dysfunction in thermal perception when tested with QST methods. However, in their comprehensive QST study on 180 patients with neuropathic pain, Rolke and coworkers [7] found differences in thermal thresholds across the body regions for the same parameters (*i.e*. WS, CS, HP and CP). They recommended that QST results have to be compared between similar body regions.

The results on orofacial pain patients showed hypersensitivity to cold stimuli on the painful side compared to the non-painful side, and for the heat stimuli these patients were less sensitive to temperature changes on the painful side compared to the non-pain side. Lang *et al*. (2005) performed a QST study on 14 patients with unilateral persistent idiopathic facial pain using cold and heat stimuli, and similar to our results, they also reported that pain patients had a higher warm sensation threshold and heat pain threshold when compared to healthy volunteers [19]. No significant change in cold sensation was described. However, their results differed to the current study as no significant difference was found in patients with unilateral pain. Langemark and his colleagues [13] undertook a study on patients with tension type headaches; they used the QST protocol on 50 patients and found that thermal pain thresholds for heat and cold stimuli were significantly different in pain patients compared to healthy volunteers. However, in contrast to our results, tension type headache patients had lower heat pain threshold and higher cold pain threshold. Interestingly in the Langemark study [13], there were significant differences in thermal thresholds between pain patients and volunteers when comparing sites that did not have any relation with headache, such as palms of the hands. These results suggest that patients with different types of orofacial pain conditions exhibit different models of thermal dysfunction when tested with QST methods.

### CONCLUSION AND LIMITATION OF THE STUDY

This study showed that QST methods using thermal stimuli could be used to evaluate sensory dysfunction in orofacial pain patients using specific parameters such as cool and warm sensation and cold and hot pain. However, reaction to thermal stimuli is diverse in different types of orofacial pain. Future studies need to be undertaken to determine sensitivity and specificity of QST for different orofacial pain conditions. Whilst the results from our study have shown interesting findings, the sample was limited to the patients attending a specific health service pain clinic. A larger study at more centres is required to assess the psychological and socio-economic factors that could play a role in pain perception.

## Figures and Tables

**Fig. (2):**
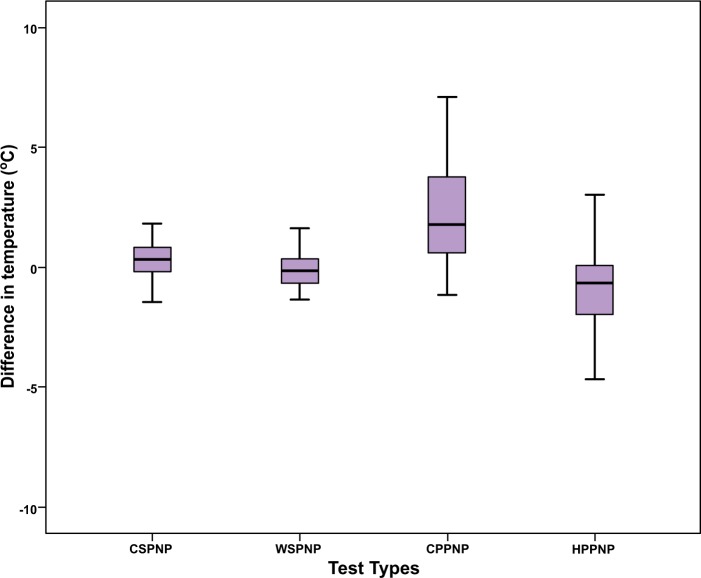
The differences in temperature (°C) between pain and non-pain sides of patients with unilateral orofacial pain using four different test types (CS, WS, CP and HP).

**Table 1:** Means and Standard Deviations of all scores of test for unilateral orofacial pain patients.

	N	Mean	Std. Deviation	Std. Error Mean
LCSPNP	60	.48 ^0^C	1.50	.194
LWSPNP	60	-.68 ^0^C	2.04	.263
LCPPNP	60	2.56 ^0^C	2.74	.354
LHPPNP	60	-1.21 ^0^C	2.59	.334

LCSPNP: Limit Cold Sensation-Pain Non Pain Side

LWSPNP Limit Warm Sensation-Pain Non Pain Side

LCPPNP: Limit Cold Pain-Pain Non Pain Side

LHPPNP: Limit Right Hot Pain-Pain Non Pain Side

## References

[1] Shy M.E., Frohman E.M., So Y.T., Arezzo J.C., Cornblath D.R., Giuliani M.J., Kincaid J.C., Ochoa J.L., Parry G.J., Weimer L.H. (2003). Quantitative sensory testing report of the therapeutics and technology assessment subcommittee of the American Academy of Neurology.. Neurology.

[2] Hansson P., Backonja M., Bouhassira D. (2007). Usefulness and limitations of quantitative sensory testing: Clinical and research application in neuropathic pain states.. Pain.

[3] Wilder-Smith O.H., Tassonyi E., Crul B.J., Arendt-Nielsen L. (2003). Quantitative sensory testing and human surgery: Effects of analgesic management on postoperative neuroplasticity.. Anesthesiology.

[4] Treede R.D., Rolke R., Andrews K., Magerl W. (2002). Pain elicited by blunt pressure: Neurobiological basis and clinical relevance.. Pain.

[5] Rolke R., Baron R., Maier C., Tölle T.R., Treede R.D., Beyer A., Binder A., Birbaumer N., Birklein F., Bötefür I.C., Braune S., Flor H., Huge V., Klug R., Landwehrmeyer G.B., Magerl W., Maihöfner C., Rolko C., Schaub C., Scherens A., Sprenger T., Valet M., Wasserka B. (2006). Quantitative sensory testing in the German Research Network on neuropathic pain (DFNS): Standardized protocol and reference values.. Pain.

[6] Dworkin R.H., Backonja M., Rowbotham M.C., Allen R.R., Argoff C.R., Bennett G.J., Bushnell M.C., Farrar J.T., Galer B.S., Haythornthwaite J.A., Hewitt D.J., Loeser J.D., Max M.B., Saltarelli M., Schmader K.E., Stein C., Thompson D., Turk D.C., Wallace M.S., Watkins L.R., Weinstein S.M. (2003). Advances in neuropathic pain: Diagnosis, mechanisms, and treatment recommendations.. Arch. Neurol..

[7] Rolke R., Magerl W., Campbell K.A., Schalber C., Caspari S., Birklein F., Treede R.D. (2006). Quantitative sensory testing: A comprehensive protocol for clinical trials.. Eur. J. Pain.

[8] Yarnitsky D., Sprecher E. (1994). Thermal testing: Normative data and repeatability for various test algorithms.. J. Neurol. Sci..

[9] Craig A.D., Bushnell M.C., Zhang E.T., Blomqvist A. (1994). A thalamic nucleus specific for pain and temperature sensation.. Nature.

[10] Lindsay KW, Bone I Neurology and neurosurgery illustrated.

[11] Yarnitsky D., Sprecher E., Zaslansky R., Hemli J.A. (1995). Heat pain thresholds: Normative data and repeatability.. Pain.

[12] Yarnitsky D., Pud D. (1997). Quantitative sensory testing.. Muscle Nerve.

[13] Langemark M., Jensen K., Jensen T.S., Olesen J. (1989). Pressure pain thresholds and thermal nociceptive thresholds in chronic tension-type headache.. Pain.

[14] Lautenbacher S., Rollman G.B., McCain G.A. (1994). Multi-method assessment of experimental and clinical pain in patients with fibromyalgia.. Pain.

[15] Kosek E., Ekholm J., Hansson P. (1996). Sensory dysfunction in fibromyalgia patients with implications for pathogenic mechanisms.. Pain.

[16] Kosek E., Hansson P. (1997). Modulatory influence on somatosensory perception from vibration and heterotopic noxious conditioning stimulation (HNCS) in fibromyalgia patients and healthy subjects.. Pain.

[17] Kosek E., Ordeberg G. (2000). Abnormalities of somatosensory perception in patients with painful osteoarthritis normalize following successful treatment.. Eur. J. Pain.

[18] Ochoa J.L., Campero M., Serra J., Bostock H. (2005). Hyperexcitable polymodal and insensitive nociceptors in painful human neuropathy.. Muscle Nerve.

[19] Lang E., Kaltenhäuser M., Seidler S., Mattenklodt P., Neundörfer B. (2005). Persistent idiopathic facial pain exists independent of somatosensory input from the painful region: Findings from quantitative sensory functions and somatotopy of the primary somatosensory cortex.. Pain.

